# Open reduction and internal fixation of acetabular fractures in patients of old age

**DOI:** 10.1007/s00264-020-04672-0

**Published:** 2020-07-30

**Authors:** Pol Maria Rommens, Roland Schwab, Kristin Handrich, Charlotte Arand, Daniel Wagner, Alexander Hofmann

**Affiliations:** 1 Department of Orthopaedics and Traumatology, University Medical Center, Langenbeckstrasse 1, 55131 Mainz, Germany; 2 Department of Orthopaedics and Traumatology, Westpfalz Klinikum Kaiserslautern, Hellmut-Hartert Straße 1, 67655 Kaiserslautern, Germany

**Keywords:** Acetabulum, Fracture, Open reduction internal fixation, Complications, Outcome, Radiologic parameters

## Abstract

**Material and methods:**

There is an ongoing debate on which treatment for acetabular fractures in elderly patients is the most appropriate. This study was set up to identify the role of open reduction and internal fixation of acetabular fractures in persons of old age.

We retrospectively reviewed the medical charts and radiological data of all patients older than 65 years, who suffered an isolated acetabular fracture and were admitted in our Department between 2010 and 2014 (5-year period). Complications, outcome and mortality were recorded. Of all surviving patients, quality of life (QoL), mobility and independence were graded with European Quality of Life 5 Dimensions 3 Level (EQ-5D-3L), European Quality of Life 5 Dimensions Visual Analogue Scale (EQ-5D-VAS), Numeric Rating Scale (NRS), Elderly Mobility Scale (EMS) and Tinetti Mobility Test (TMT).

**Results:**

Seventy patients could be identified. There were 52 men (74%) and 18 women (26%) with a median age of 79.0 years (range: 65–104 years). Forty-six patients (66%) had been treated with open reduction and internal fixation (ORIF), 24 (34%) conservatively. There were negative predictive factors—subchondral impaction, damage to the femoral head and multiple fragments—in 54% of the operative group. With ORIF, an anatomical reduction could be achieved in 27 patients (59%), an acceptable in 18 (39%) and a poor in one (2%). At follow-up, 18 patients (26%) had died and 23 (33%) were not able to participate. The follow-up rate of the surviving operatively treated patients was 77%. Eleven of 46 operated patients (24%) needed a conversion to a total hip arthroplasty (THA). All patients undergoing conversion had imperfect reduction after surgery. No patient in the non-operative group underwent conversion to THA during follow-up. The median follow-up time of operatively treated patients without conversion (*n* = 17) was 30 months (range, 16–73 months), of patients with THA (*n* = 9) 30 months after conversion (range, 17–55 months). Quality of reduction correlated to QoL, mobility and independence in all recorded parameters. Patients with secondary THA had similar good outcomes as patients after ORIF without later conversion. Men had better outcome than women.

**Conclusion:**

ORIF of acetabular fractures in patients of old age results in excellent outcomes at short-term follow-up when anatomical reduction can be achieved. In case of negative predictive factors, ORIF cannot be regarded as a definitive solution, rather as the construction of a stable socket for secondary THA. The decision of therapy should be made dependent on pre-operative radiographic parameters.

## Introduction

Acetabular fractures are often the result of a high-energy trauma such as a traffic accident or a fall from a great height [1–4]. Yet, we are increasingly confronted with fractures due to low-energy trauma. The percentage of persons older than 65 years has grown from 16 to 21% between 1994 and 2019 (25-year period) in the German population (https://de.statista.com/themen/172/senioren/). Consequently, the incidence of acetabular fractures in patients above 65 years has grown as well [5–8]. Today, the majority of the acetabular fracture patient population is constituted by patients of old age [9]. This raises the question of the role of open reduction and internal fixation (ORIF) of acetabular fractures in this patient population. In the study of Tannast et al., age of more than 40 years was an independent predictive factor for conversion to total hip arthroplasty (THA) after ORIF [10]. In older patients, treatment alternatives are not clearly supported by evidence. Long-term outcome studies are rare [11, 12]. Old patients have different characteristics: most have comorbidities with increased operative risk. Functional demands are lower. Bone quality is diminished, which bears the risk of implant loosening. On the other hand, elderly patients should be mobilised timely after trauma. Restricted weight bearing may not be possible. Non-operative treatment has the disadvantage of delayed and painful mobilisation. Joint congruence is not obtainable unless the fracture is not displaced or shows secondary congruence. Some authors prefer non-operative management in specific conditions [13, 14]. Stable cup fixation in primary THA may be challenging in displaced fractures [15]. Due to low bone quality and subchondral impaction, anatomical reduction may not be possible in ORIF [16]. Other solutions as ORIF followed by THA in one or consecutive operation phases are presented, but not supported by large series [17–19]. This study aims at describing the short-term outcomes of ORIF of acetabular fractures in a cohort of patients of old age. Outcomes are evaluated with regard to morphologic criteria in conventional X-rays and CT data of the fracture and with validated questionnaires.

## Patients and methods

We retrospectively reviewed the medical charts of all patients aged 65 years or older with an isolated acetabular fracture, admitted to our level I trauma centre between 2010 and 2014 (5-year period). Approval of this retrospective study has been granted by the local ethics committee (Ethics Commission of the State Chamber of Medicine of Rhineland-Palatinate) (Ref. 837.140.17 (10974)). Polytraumatized patients, acetabular fractures due to malignancies, periprosthetic acetabular fractures and patients with follow-up to less than one year were not included. Demographic data, type of fracture (by the Letournel-classification [20]), type of treatment (operative or non-operative), radiographic result (by the Matta criteria [21]) and perioperative complications were recorded. The following negative outcome predictors were identified on pre-operative radiographs and CT: subchondral impaction, damage to the femoral head and fracture comminution [22]. Date of death was registered for patients who died. All surviving patients were asked to fill in the European Quality of Life 5 Dimensions 3 Level (EQ-5D-3L) questionnaire [23, 24] and the numeric rating scale (NRS) [25]. The timed up-and-go test (TUG) [26], the elderly mobility scale (EMS) [27] and the mobility test of Tinetti (TMT) [28] were performed in all patients with follow-up by one of the co-authors (SR).

Statistical analysis was performed using the SPSS software (IBM SPSS Statistics for Windows, Version 25; IBM Corp, Armonk, NY, USA). A descriptive analysis of demographics, fracture type, therapy inclusive surgical approaches, length of hospital stay, complications, mortality, functional outcome and degree of independence was performed. We further performed a comparison of the treatment results depending on the gender and type of treatment. To ascertain normal distribution, we utilised the Shapiro-Wilk Test. The PRO EQ-5D VAS was normally distributed (*p* = 0.076); the remaining PRO EQ-5D Index Value was not (*p* = 0.00026). *T* test was used in case of normal distribution and variance homogeneity. In case of variance homogeneity without normal distribution, the Mann-Whitney *U* test was used (e.g. to calculate the significance between the EQ-5D Index Value and the gender). When more than two groups were compared, unifactorial ANOVA test was used in case of normal distribution and variance homogeneity. If there was normal distribution without variance homogeneity, we performed the Welch-test. The Kruskal-Wallis test was used in case of variance homogeneity without normal distribution (e.g. the EQ-5D Index Value depending on the quality of reduction). All multivariate tests included a post hoc analysis. A statistically significant difference was present when *p* < 0.05. The Kaplan-Meier estimate was used for the presentation of the patient survival rate and for the survival rate of hip joints with or without anatomical reduction.

## Results

Seventy patients were identified. There were 52 men (74%) and 18 women (26%). Their median age was 79 years (range, 65–104 years), 8 patients being more than 90 years old. A total of 46 patients (66%) had been treated with ORIF, 24 (34%) had been treated non-operatively. The median age of operatively treated patients was 77 years (range, 65–92 years), of conservatively treated patients 81 years (range, 73–104 years). Seven of eight patients above the age of 90 had been treated conservatively. During the follow-up period, 18 patients (26%) had died, 12 (26%) from the operatively treated group and 6 (25%) from the conservatively treated group. A total of 23 patients (33% of the whole group or 44% of the survivors) refused participation in the study or were not able to participate (mental illness, loss of mobility): 8 of the operatively treated group (8/34 = 23%) and 15 of the conservatively treated group (15/18 = 83%). There were 29 patients available for the follow-up examinations, 26 from the operative and 3 from the conservative group. The follow-up rate of the surviving operatively treated patients was 77%. Median time to follow-up was 36 months (range, 16–73 months). Demographics are depicted in Table 1.

**Table 1:** Demographic data

	Total	Operative	Conservative
Number of patients (%)	70 (100)	46 (100)	24 (100)
Age in years: median (range)	79 (65–104)	77 (65–92)	81 (73–104)
Mortality: no (%)	18 (26)	12 (26)	6 (25)
	No (%)	No (%)	No (%)
Surviving patients	52 (100)	34 (100)	18 (100)
Drop-out of surviving	23 (44)	8 (23)	15 (83)
Remaining for evaluation	29 (56)	26 (77)	3 (17)

Fractures involving the anterior column and anterior wall represented 90% of the whole group and 89% of the operatively treated group with complete follow-up (Table 2).

**Table 2:** Classification of fractures

	All study patients	Follow-up patients with operative treatment
	No (%)	No (%)
All patients	70 (100)	26 (100)
Anterior column, posterior hemitransverse	26 (37)	12 (46)
Both column	25 (36)	7 (27)
Anterior column	10 (14)	4 (15)
Anterior wall	2 (2.9)	-
Posterior wall	2 (2.9)`	1 (3.8)
Posterior column	2 (2.9)	-
T-type	2 (2.9)	2 (7.7)
Posterior column, posterior wall	1 (1.4)	-

More than 90% of operatively treated patients and more than 95% of the operated patients with complete follow-up were operated through one of the anterior approaches (Table 3).

**Table 3:** Choice of approaches

	Operatively treated patients	Follow-up patients with operative treatment
	No (%)	No (%)
All patients	46 (100)	26 (100)
Ilioinguinal	36 (78)	22 (85)
Kocher-Langenbeck	4 (8.7)	1 (3.8)
Intra pelvic and lateral window	3 (6.5)	2 (7.7)
Intra pelvic	2 (4.3)	1 (3.8)
Iliofemoral	1 (2.2)	–

The median length of hospital stay of all patients was 14 days, in the operatively treated group 16 days and in the conservatively treated group 8 days. Complications occurred in 26% of patients in the operative and 38% in the conservative group (*p* = 0.32) (Table 4).

**Table 4:** In-hospital complications

Total	Operative	Conservative
	No (%)	No (%)
Number of patients	46 (100)	24 (100)
Pneumonia	7 (15)	6 (25)
Urinary tract infection	4 (8.7)	5 (21)
Decubitus	3 (6.5)	1 (4.2)
Deep wound infection	1 (2.2)	-
Pulmonary embolism	1 (2.2)	-

**Fig. 1:**
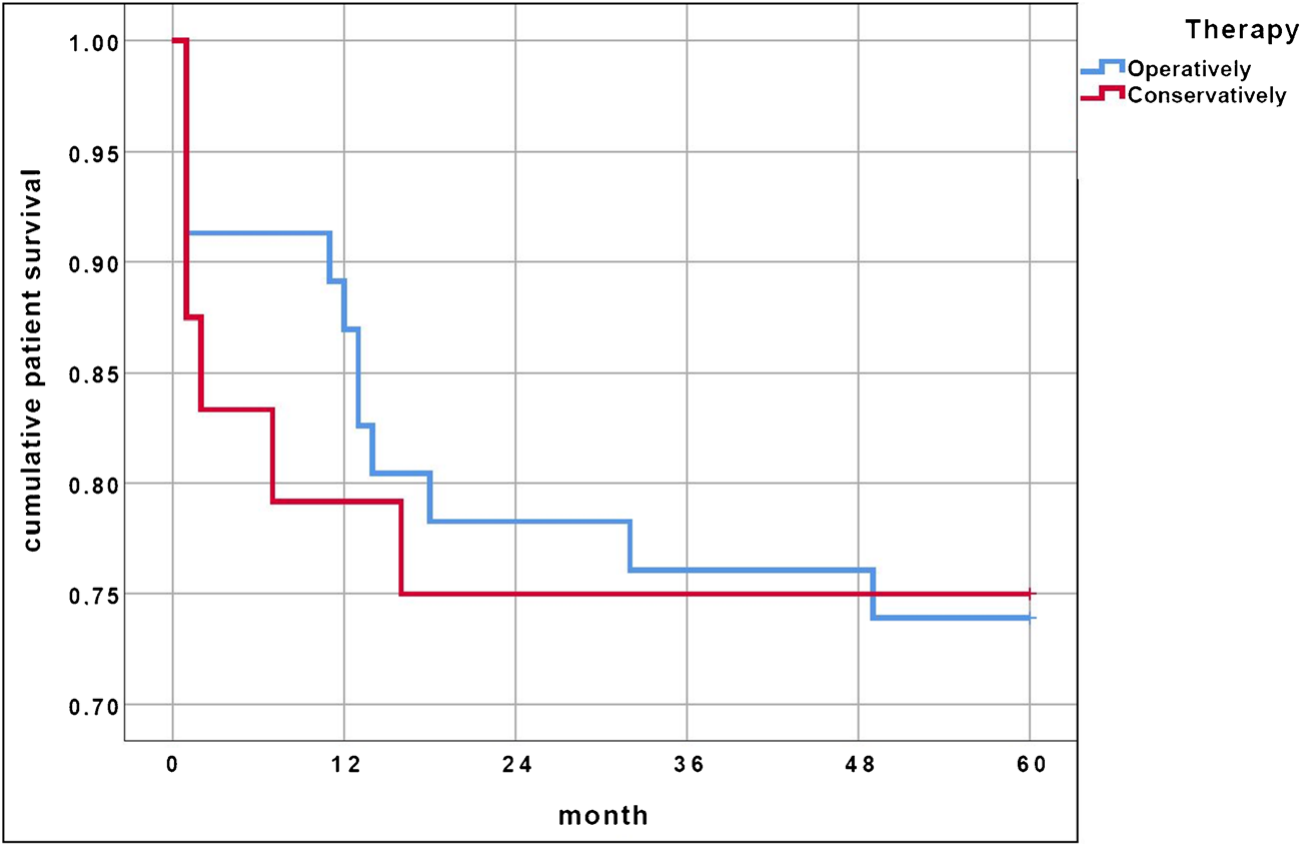
Kaplan-Meier curves of cumulative patient survival during 5-year follow-up depending on type of treatment

Mortality during 5-year follow-up was documented for the entire cohort. There was a 30-day mortality of 9%, 7% in the operative group and 13% in the conservative group. The one-year mortality was 14%: 11% in the operative group and 21% in the conservative group. The five-year mortality was 26%, 26% in the operative and 25% in the conservative group. The Kaplan-Meier curves for cumulative patient survival of the operative and conservative groups are depicted in Fig. 1.

**Fig. 2:**
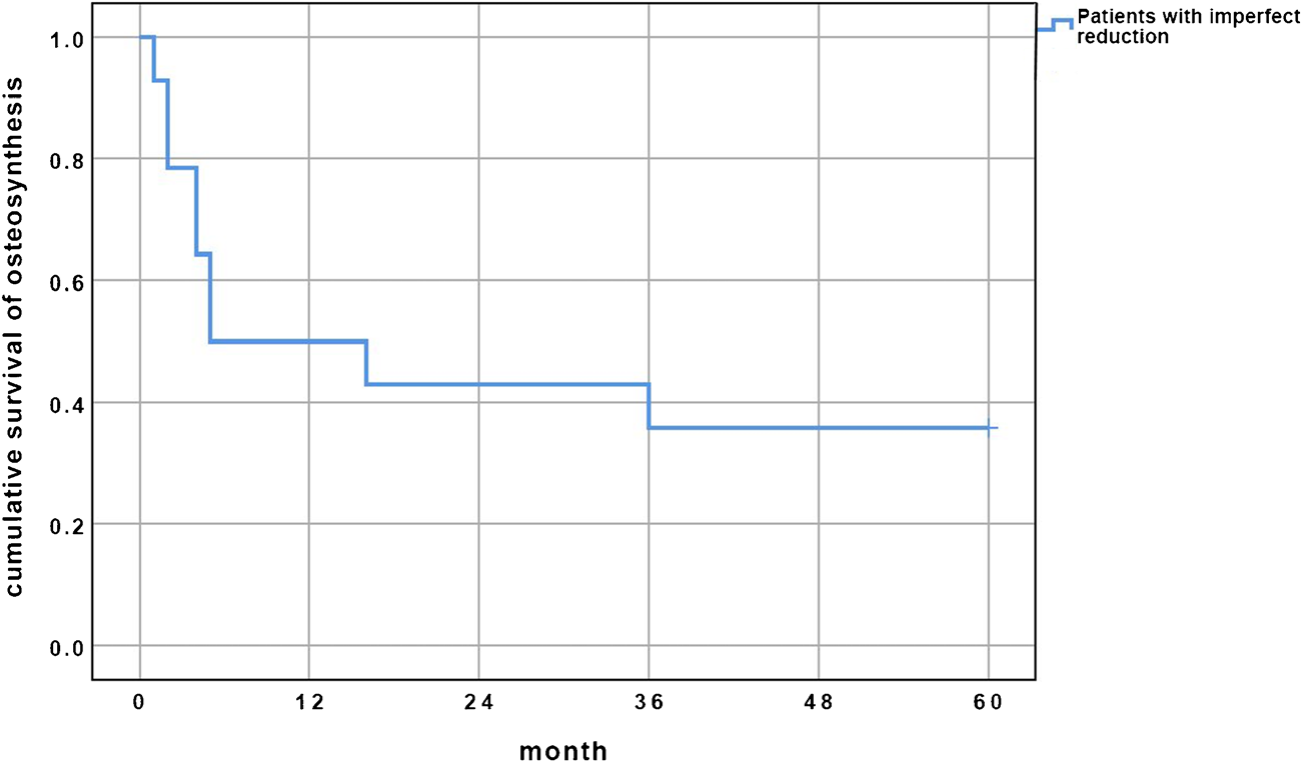
Kaplan-Meier curve of cumulative hip joint survival during 5-year follow-up of the patients with imperfect and poor reduction. In 27 patients with anatomical reduction, no secondary THA was performed

Negative predictive factors were present in 21 patients (46%) of the whole operative group. An anatomical reduction could be achieved in 27 patients (59%). Further details on type and number of negative predictive factors and on the quality of reduction can be seen in Table 5. During follow-up, 11 patients (16%) received a THA. No patient with conservative therapy received THA. The ratio of conversion to THA in the operated group was 24% (*n* = 11/46). All patients, who needed conversion, had an imperfect reduction. There was no conversion in patients with anatomical reduction. There were 3 reasons for conversion to secondary THA. Four patients had implant loosening with secondary fracture displacement. Their conversion took place after one, two, two and four months respectively. One patient had an intra-articular screw with consecutive destruction of the femoral head. THA was performed after five months. Six patients developed a post-traumatic hip osteoarthritis. THA was carried out after four, four, five, 11, 16 and 36 months respectively. The Kaplan-Meier curve of cumulative hip joint survival in patients, who had imperfect reduction after ORIF, is depicted in Fig. 2.

**Table 5:** Negative predictive factors and quality of reduction

	Operated patients	Follow-up patients with operative treatment
	No (%)	No (%)
All patients	46 (100)	26 (100)
Gull sign	7 (15)	5 (19)
Additional subchondral impaction	6 (13)	5 (19)
Indentation of femoral head	6 (13)	3 (12)
Fragment comminution	2 (4.3)	1 (3.8)
Anatomical reduction	27 (59)	11 (42)
Not anatomical, acceptable	18 (39)	14 (54)
Poor reduction	1 (2.2)	1 (3.8)

The mean EQ-5D Index Value was 0.82 (SD 0.22) for the group with complete follow-up. The EQ-5D Index Value was 0.80 (SD 0.20) for the age group between 65 and 74, and 0.84 (SD 0.24) for the age group of 75 and above. The median EQ-5D Index Value was 0.95 (range 0.31–1.0, IQR 0.89–1.0) for the patient group with anatomical reduction, 0.79 (range 0.40–1.0, IQR 0.72–0.89) for the group with imperfect reduction and 0.11 for the patient with poor reduction (*p* = 0.029). Men had a higher median EQ-5D Index Value of 0.89 (range 0.31–1.0, IQR 0.79–1.0) than women with a value of 0.70 (range 0.11–0.90; IQR 0.48–0.79) (*p* = 0.016).

The mean EQ-5D VAS was 66 (SD 14) for the whole group with complete follow-up. It was higher in patients with anatomical reduction (71.1; SD 9.2) than in patients with imperfect and poor reduction (61.5; SD 16.3). Men had a higher EQ-5D VAS (69.9; SD 11.6) than women (51.5; SD 13.7) (*p* = 0.002).

Median intensity of pain in the NRS of the whole group with complete follow-up was 2. Patients with anatomical reduction reported a median pain intensity of 2; patients with an imperfect reduction reported a median pain intensity of 3 (*p* = 0.018). Women reported a median pain intensity of 4 compared with men with a median of 2 (*p* = 0.004).

For the TUG, patients with anatomical reduction needed 17 s (SD 7 s), whereas patients with imperfect reduction needed 24 s (SD 11 s) (*p* = 0.057). Men needed 17 s, which was significantly less than women with 34 s (*p* < 0.001). The median EMS of patients with complete follow-up was 17 points. Patients with anatomical reduction reached a mean EMS value of 17 (SD 2.5), whereas patients with imperfect reduction a value of 15 (SD 3.9) (*p* = 0.223). Men had 18 points, which was significantly better than women, who had 12 points (*p* = 0.04). The median TMT was 23.3 points, which corresponds with a moderate risk of falling. Patients with an anatomical reduction had a mean TMT value of 27, patients with an imperfect reduction a value of 25 (*p* = 0.27). Higher values were recorded for men with 27 points than women with 15 points, which means that women had a higher risk of falling than men (*p* = 0.011).

When comparing patients with osteosynthesis as only treatment (*n* = 17) with patients, who received a conversion to THA (*n* = 9), all parameters were slightly better for patients without conversion, but the differences were not statistically significant. The mean EQ-5D Index Value for the patients without conversion was 0.82 (SD 0.25), for the patients with conversion 0.75 (SD 0.15). The median EQ-5D VAS for the patients without conversion was 70 (35–85, IQR 65–76), for the patients with conversion 62 (35–87, IQR 50–64). Further comparative data can be seen in Table 6.

**Table 6:** Comparison between patients without and with conversion to total hip arthroplasty

	Patients without conversion (*n* = 17)	Patients with conversion (*n* = 9)
Median follow-up time in months (range)	30 (16–73)	39 (30–60)
Median follow-up time after conversion in months (range)	-	30 (17–55)
EQ-5D Index Value, mean (SD)	0.82 (0.25)	0.75 (0.15)
EQ-5D VAS, median (range, IQR)	70 (35–85, 65–76)	62 (35–87, 50–64)
NRS, mean (SD)	2.3 (1.5)	3.4 (19)
TUG, mean (SD)	18.7 (7.4)	24.9 (12.6)
EMS, mean (SD)	17 (2.5)	14.8 (4.6)
TMT, mean (SD)	24.7 (3.7)	20.8 (7.3)

## Discussion

We retrospectively reviewed 70 patients of old age with acetabular fractures. Nearly three quarters of patients were men. In a study on fragility fractures of the pelvis performed in the same level I trauma centre, more than 80% were women [29]. The loss of bone strength in the pelvic ring while ageing may be different in women and men [30, 31]. Patients, who were treated conservatively (median age, 81 years), were significantly older than patients treated operatively (median age, 77 years). Operative risk, functional demands and life expectancy played an important role in decision-making.

Mortality and drop-out ratios in our patient cohort during follow-up time reflect data of other studies with patients of similar age such as hip fracture patients [32]. Only three non-operatively treated patients completed follow-up, meaning that no conclusions can be drawn about outcomes in the non-operative group. The 77% follow-up rate of operatively treated patients is fair. The median follow-up time of the operated group was 36 months (range, 16–73 months). A total of 23 of the 26 operated patients with follow-up had a follow-up time of 24 months or more, which is considered enough to detect most cases of osteoarthritis after acetabular fractures [33].

While analysing the pre-operative conventional radiographs and CT data, we found negative predictive factors in 45% of the operated fractures. These factors had a major influence on the accuracy of reduction. An anatomical reduction could only be obtained in 59%. The quality of reduction played a decisive role for QoL, functional outcome and necessity of conversion to THA. The EQ-5D Index Value and the EQ-5D VAS were significantly higher in the patients with anatomical reduction than in patients with imperfect reduction. The mean EQ-5D Index Value was 0.95 for the patient group with anatomical reduction and 0.79 for the group with imperfect reduction. For comparison, these values are 0.89 for a previously described German reference population between 65 and 74 years of age and 0.84 for a population older than 75 years [34]. The mean EQ-5D VAS of patients with anatomical reduction was 71.1 and of the patients with imperfect reduction 61.5. Reference values rank between 60 (women above 75 years of age) and 72 (men between 65 and 74 years of age) in a previously described German reference population [35]. Pain intensity (NRS) was graded significantly higher in patients with imperfect reduction. Patients with an anatomical reduction needed 17 s for the TUG test, whereas patients with an imperfect reduction needed 24 s. The only available reference value for the TUG test gives a much shorter time of 9.2 s for the age group between 70 and 79 years [35]. The median EMS of patients with complete follow-up was 17 within a maximum of 20 points. As values above 14 stand for independent mobilisation, we can conclude that the majority of patients were independent. Nevertheless, women with a mean value of 12 points scored significantly lower than men. During follow-up, 24% of the operated patients needed a conversion to THA. All of them had negative predictive factors, imperfect reduction or complications of surgery. There was no conversion in patients with anatomical reduction, which also has been described in previous studies [10, 21, 36].

There were more in-hospital complications in the conservative group than in the operated group (38% versus 26%) although the hospital stay of the conservative group was shorter (8 days versus 16 days). Mortality within the first 30 days after trauma was nearly double in the conservative group, but the study did not have statistical power to analyse mortality. Mortality within the first year was still higher in the conservative group but became similar after five years for both groups. The early period after trauma seems to be more critical for the conservative group for what concerns complications and mortality. The higher age of conservatively treated patients may play a decisive role here.

Previous studies on the treatment of acetabular fractures in patients with old age have been published. Helfet et al. presented a series of 18 patients with an average age of 67 years, who had been treated with ORIF. Patients reached an average Harris Hip Score (HHS) of 90 points after two years. The authors concluded that ORIF can obtain good results and avoid the need for a difficult THA in selected patients [36]. Carroll et al. reviewed 93 patients with a mean age of 67 years and a follow-up of five years. The rate of secondary THA was 31%. Poor fracture reduction, development of avascular necrosis and previous contralateral THA were associated with secondary surgery. Functional outcomes were similar to those of younger patients with acetabular fractures and with the non-injured norms [12]. Jeffcoat et al. presented 41 patients with a minimum of two years follow-up. Conversion to THA was needed in 27% [37]. Rickman et al. presented 12 patients, in which a combination of ORIF and THA were performed in the same operation. Patients were allowed full weight bearing immediately. Outcomes were favourable and complications few. No cup migration was seen after a mean time of 18 months [38]. Mouhsine et al. presented cable fixation in combination with THA. Eleven of 12 patients could be followed up for two years. They had complete fracture healing and good functional outcomes without cup loosening [39]. Salama et al. presented a series of 18 patients with ORIF and THA in one operative procedure. They had an average age of 60 years and a mean follow-up of 22 months (range, 12–36 months). All fractures healed and there was cup loosening in only 1 patient [40]. Borajah et al. reviewed 18 patients with a mean age of 71 years and at least one year follow-up. They all received ORIF and THA in one operative session. There was one failure of the acetabular component. Mean HHS was 88 [41]. Borg et al. also suggest performing open reduction and internal fixation together with THA in one operative session. No patient of their cohort of 13 patients, who underwent this combined procedure, needed secondary surgery within three years of follow-up [19]. The strategy of combining ORIF with THA may decrease the need for secondary surgery, but may also add to surgical time at the initial operation. Sermon et al. reviewed 121 patients, who received THA after an acetabular fracture. They differentiated between patients who received immediate THA (early THA group) and those who received THA after previous ORIF (late THA group). The revision rate after THA was 22% in the late THA group. This rate was significantly higher than the revision rate of 8% in the early THA group. Patients with primary THA were significantly older than patients with secondary THA [42]. De Bellis et al. performed a systematic review of 6 studies concerning THA after acetabular fracture. Similar satisfying clinical outcomes were seen after acute or delayed THA [43]. Our results also show similar functional outcomes in patients after ORIF and later conversion to THA, when compared with patients after ORIF only. In our concept, delayed THA aims at safe placement of the cup in a stable and healed acetabular socket.

All abovementioned publications report on small cohorts, with retrospective analysis, and short follow-up times. Our study also has all these limitations. Moreover, the follow-up time of 36 months of our operated group was short. With longer follow-up time, the rate of conversion to THA may have increased. Although, with a median age of 77 years in the operated group, our study reports on a patient population of old age.

## Conclusion

In the absence of negative prognostic radiographic signs, ORIF of acetabular fractures in patients of old age is feasible and yields very good functional outcomes at short-term follow-up. Besides age and health status, decision-making should be based on the radiological characteristics of the fracture. In case of negative predictive factors, ORIF can be regarded as a way of reconstructing a stable socket for later safe cup implantation.
